# Examining ethno-racial attitudes of the public in Twitter discourses related to the United States Supreme Court *Dobbs vs. Jackson Women's Health Organization* ruling: A machine learning approach

**DOI:** 10.3389/fgwh.2023.1149441

**Published:** 2023-05-04

**Authors:** Otobo I. Ujah, Pelumi Olaore, Onome C. Nnorom, Chukwuemeka E. Ogbu, Russell S. Kirby

**Affiliations:** ^1^ College of Public Health, University of South Florida, Tampa, FL, United States; ^2^ Department of Community Medicine, Jos University Teaching Hospital, Jos, Nigeria

**Keywords:** *Roe vs. Wade*, racism, race, ethnicity, sentiment analysis, structural topic modeling, natural language processing, social media

## Abstract

**Background:**

The decision of the US Supreme Court to repeal *Roe vs. Wade* sparked significant media attention. Although primarily related to abortion, opinions are divided about how this decision would impact disparities, especially for Black, Indigenous, and people of color. We used advanced natural language processing (NLP) techniques to examine ethno-racial contents in Twitter discourses related to the overturn of *Roe vs. Wade*.

**Methods:**

We screened approximately 3 million tweets posted to *Roe vs. Wade* discussions and identified unique tweets in English-language that had mentions related to race, ethnicity, and racism posted between June 24 and July 10, 2022. We performed lexicon-based sentiment analysis to identify sentiment polarity and the emotions expressed in the Twitter discourse and conducted structural topic modeling to identify and examine latent themes.

**Results:**

Of the tweets retrieved, 0.7% (*n *= 23,044) had mentions related to race, ethnicity, and racism. The overall sentiment polarity was negative (mean = −0.41, SD = 1.48). Approximately 60.0% (*n *= 12,092) expressed negative sentiments, while 39.0% (*n *= 81,45) expressed positive sentiments, and 3.0% (*n *= 619) expressed neutral sentiments. There were 20 latent themes which emerged from the topic model. The predominant topics in the discourses were related to “racial resentment” (topic 2, 11.3%), “human rights” (topic 2, 7.9%), and “socioeconomic disadvantage” (topic 16, 7.4%).

**Conclusions:**

Our study demonstrates wide ranging ethno-racial concerns following the reversal of *Roe* and supports the need for active surveillance of racial and ethnic disparities in abortion access in the post-*Roe* era.

## Introduction

Abortion in the United States (US) has remained a subject of longstanding ethical, religious and political controversy (1). As far back as the early 19th-century abortion was considered legal and by the mid-19th century, at least one in four pregnancies ended in abortion (2). However, by the 20th century, abortion restrictions had grown across several states in the US which consequently heralded an era of illegal and unsafe abortions that contributed to about 17% of maternal mortality in the US (2). While limited access to safe abortion services remained available for socially advantaged women, young women and those from minority populations continued to be disproportionately affected by having recourse to unsafe abortions (3). The increase in unsafe abortions as well as an increase in Thalidomide-associated birth defects strengthened further advocacy efforts to decriminalize abortion (2). Eventually, in 1973, the United States Supreme Court in a landmark decision in *Roe vs. Wade* ruled that the decision to terminate or continue a pregnancy was the constitutional right of women under the protection of the “right to privacy” (4). Under this legal framework, states were prohibited from restricting abortion before the third trimester of gestation (5, 6). Beyond this point, however, states were allowed to regulate abortion except when the woman's life was at risk. Since then, there have been several legislative barriers that have confronted abortion access, and data from survey polls show a roughly even split of the US population into those opponents and supporters of abortion (7).

In the 1992 *Planned Parenthood* *vs*. *Casey* decision, the Supreme Court upheld the constitutional right to abortion but permitted states to restrict abortion before 24 weeks of gestation (6). However, the Supreme Court ruling in *Dobbs vs. Jackson Women's Health Organization* regarding the constitutionality of a Mississippi law banning abortion after 15 weeks of pregnancy led to the eventual overturn of *Roe vs. Wade*, putting an end to the 49 years 5 months and 2 days constitutional right to abortion in the United States and consequently, returning the authority to regulate abortion to individual states (8–10). Since *Dobbs*, there have been varying nationwide implementation of bans or protection of abortion access (11) with several states imposing partial or complete abortion restrictions. In conjunction with a woman's location of residency, restrictive abortion laws serve as socio-ecological determinants of abortion care access which can potentially impact her sexual and reproductive health and well-being (12–14). The reversal of *Roe vs. Wade* has generated substantial concerns among the public. However, there are some who find the decision favorable within the context of the morality of abortion. These individuals may believe that abortion is morally wrong and that the reversal of Roe vs. Wade is a positive step in the right direction (15). Those in favor of the Supreme Court's decision also argue that the decision is likely to result in a decrease in the number of abortions performed and ultimately increasing the sanctity of life.

With prior state-level abortion restrictions, some of the challenges encountered with accessing abortion care and services include delays in care, facility closures, long-distance travels for women seeking abortion, and increased cost for abortion services (6, 16) which were associated with medical, economic and safety problems that are likely to be disproportionately worse, especially for Black, Indigenous, and other people of color (BIPOC) in the post-*Roe* landscape (16–18). Furthermore, there is evidence of racial and ethnic differences in abortion rates and access in the United States, with the need for abortion services being greatest among women of color—Additionally, they face more obstacles to abortion treatment than other racial and ethnic groups (4, 15). Although Dobbs *was* primarily related to abortion, however, opinions are divided about how the decision impacts the status of federal right to contraception, consensual sexual intimacy, marriage, and reproduction (14) as well as to what extent the Supreme Court's decision is likely to exacerbate extant health disparities, especially for women of color who, compared to White women are likely to have lower income (4, 19). Stevenson (20) estimates that with a total ban in effect, overall maternal mortality is likely to rise by approximately 21% with the rates being even higher for Black women (33%).

According to the theory of planned behavior, attitude and opinion together with perceived control and contextual subjective norms shapes behavioral intention and subsequent behavior (21, 22). Therefore an understanding of these opinions could inform strategies and help gain insight into salient issues that can inform future research, policy, and practice related to eliminating racial and ethnic disparities. While public opinion polls and national surveys have been employed in assessing different dimensions of attitudes towards abortion, these approaches are however limited by how questions used to collect data are framed, by repetition of questions on complex issues which exhibit a temporal trend, and by the failure to capture robust information of public sentiments (23, 24). There is a growing interest in the use of social media, particularly Twitter, as a unique source of big data for public health research, since it provides real-time content and is easily accessible and searchable (25–27). Furthermore, relative to traditional approaches to data collection which tend to be expensive and time-consuming, social media as a data source is efficient and cost-effective for data collection, recruitment of study participants, and delivery of interventions (2, 21). Also, due to the evolving nature of social media discourses, it helps provide opportunities for researchers to discover new, and previously unidentified perspectives (28). Additionally, there is a substantial quantity and scope of data from Twitter, which represents a variety of user demographics (25). Considering that abortion is one of the frequently discussed issues on social media platforms (27) and several studies have used Twitter to examine abortion opinions (2, 4, 21, 27), women who may potentially seek and eventually access abortion services, especially with the post-*Roe* landscape may rely on information from social media and shape discourses about their experiences through this platform. Therefore, as an important source of data, Twitter can provide an opportunity for early and rapid assessment of the public's concerns about abortion restriction which lays the foundation for the design and implementation of future research and programs.

An inherent challenge with the use of big data from social media for qualitative research arises from the need for manual processing and analysis of large volumes of unstructured texts in a systematic and reproducible manner (29). It frequently results in premature sampling and early selection of focal texts, problems integrating perspective and interdiscursivity (29). Nevertheless, this challenge can be overcome by employing advanced natural language processing and computational text analytic methods such as sentiment analysis and topic modeling to uncover attitudes and salient themes emerging from public discourses contained in big data (29, 30). Several studies have employed computational text analytic methods to examine population attitudes toward contraception (31), topics and sentiments expressed in health events (32), abortion legislation (24, 33, 34), including the recent Supreme Court ruling in *Dobbs* (2), COVID-19 pandemic (35) and “Black Lives Matter” (36). Also, the utility of Twitter in studying abortion attitudes draws from existing literature which showed that opponents and proponents of abortion, including public figures and social movement organizations use their social media platforms to demonstrate their solidarity, spread information, mobilize supporters and raise funds for events following the referendum repealing Eight Amendment of the constitution in Ireland in 2017 (37). Therefore, considering the public's reaction to the overturning of *Roe vs. Wade* on social media, Twitter can help play an important role in evaluating public knowledge and concerns regarding restrictions on abortion access and as well, can inform how contextually appropriate strategies to address these concerns are designed and implemented.

Data mining techniques such as those in natural language processing are effective for representing the opinions of pro-abortion and anti-abortion supporters in the context of the reversal of *Roe vs. Wade*. A recent study by Mane, Yue (2) examined the public's reaction on Twitter to the overturning of *Roe vs. Wade* and abortion bans. The authors discuss spatial, thematic, and sentiment patterns in Twitter content on the abortion ban before and after *Dobbs*. Given substantial concerns about how the Supreme Court's decision may disproportionately impact BIPOC, there is a need to understand how the overturning of *Roe* is perceived and interpreted in the context of racial and ethnic inequalities by the general public. By employing advanced natural language processing (NLP) techniques, this study examined ethno-racial attitudes in Twitter discourses related to the overturn of *Roe vs. Wade*.

## Methods

The steps involved to address the research questions for this study include data acquisition, data cleaning and preprocessing, and data analyses. Each step was performed using R software version 4.2.0.

### Data acquisition

Data for this study were collected from Twitter, which has more than 300 million active users monthly. A random sample of original tweets posted in English language between June 24 (Supreme Court ruling) and July 10, 2022, were collected through Twitter's official application programming interface (API) using the “*twitteR*” package in R. This period was chosen with the goal of capturing discourses in the early phase of the reversal of *Roe vs. Wade* and therefore likely to reflect important concerns of the public. Initially, tweets related to the overturn of Roe vs. Wade were retrieved using related keywords/phrases and hashtags which included “*Roe*”, “*Roe vs. Wade*”, “*roevswade*”, “*roeoverturned”, “Roe* vs. *Wade*”, “#*Roe*”, and “#*roevwade*”. Retweets were, however, excluded from the retrieved data since they do not contain as much thought as an original tweet and can serve as a source of bias in the data (38). To filter tweets for mentions of racism and different racial and ethnic categories, we customized and revised the filter terms from existing literature (39). These included “*black men”, “black women”, “black people”, “white men”, “white women”, “white people”, “hispanic men”, “hispanic women”, “hispanic people”, “latino”, “latina”, “latinx”, “asian men”, “asian women”, “asian people”, “men of color”, “women of color”, “people of color”, “bipoc”, “racial”, “racism”, “blacks”, “Hispanics”, “whites”, “Caucasian”, “Black American”, “African American”.* Tweets used in this study are publicly available. Permission to use data was obtained from Twitter developers prior to data collection hence, ethical approval was not required. However, in order to maintain privacy of user accounts, mentions were replaced with “*@username”*.

### Data cleaning and preprocessing

Prior to performing data analyses, dimensionality reduction in terms of data cleaning and preprocessing techniques was performed. This involved replacing contractions, removing special characters (“*&*”, “*@*”, “*$*”,“*#*”), numbers, account usernames, non-American Standard Code for Information Interchange (non-ASCII) characters from strings, hyperlinks, white spaces, emojis, punctuations, sentence breaks, duplicate tweets and stop words. Also, texts were converted to lower case, and tweets containing four or fewer words were excluded from the corpus of tweets given that they do not provide useful semantics. Lastly, tokenization of texts into single words was performed.

### Data analyses

We employed natural language processing (NLP) techniques—sentiment analysis and topic modeling—to address the research questions for this study. Both techniques are useful for providing nuanced insight into discourses in unstructured texts such as in user-generated content from social media. Sentiment analysis applies computational linguistics to identify and assess opinions and attitudes about events contained in textual data (40). This could be lexicon-based, machine learning based, or a hybrid of both methods. In addition, sentiment analysis could be performed at the text, word, or document level. This study applied the “*syuzhet*” package to conduct a lexicon-based sentiment analysis (41). This algorithm uses normalized scores to classify texts, based on sentiment polarity, into either positive, negative, or neutral sentiments. In addition, this package uses the Canadian National Research Council's (NRC) Word Emotion Association Lexicon to classify emotions within the corpus of text in to anticipation, anger, joy, surprise, trust, disgust, fear and sadness according to Plutchik's human emotion classification. In performing sentiment analysis, the term “abortion” was excluded from the corpus since most lexicons score abortion highly negatively thereby biasing the output. In addition, the use of the abortion was more in reference to the news event rather than an individual receiving the procedure.

Topic modeling, on the other hand, is a form of unsupervised machine learning technique employed to detect word clusters which frequently co-occur within unstructured data such as social media data. In this study, structural topic modeling (STM) with spectral initialization was performed to identify latent themes (42). The STM is an extension of the Latent Dirichlet Al.location (LDA) which integrates features of a correlated topic model and sparse additive generative topic model. The “*stm*” package was applied to run the topic modeling (43). Using the *searchK()* function, we trained multiple STM models with different numbers of topics ranging from 5–50 in increments of 5 and evaluated the coherence-exclusivity plot to identify models best potential candidate topics. In addition, we iteratively compared output from the model diagnostics and selected the model with a high semantic coherence, high held-out likelihood, high exclusivity, and low residual based on recommendation from prior studies (44, 45). By examining tables and plots of the semantic coherence and exclusivity estimates, the model selection for the number of topics was based on a trade-off between the semantic coherence and exclusivity of a model in which there was no dominance by either metric (43). The researchers examined the 10 most frequently used words in each topic to assign a label and interpreted based on highest probability, FREX, score, and lift metrics (46). Each topic was then classified based on sentiment polarity into topics with positive and negative sentiments. Furthermore, we performed a correlation network analysis to examine the correlation between the topics.

## Results

**Figure 1:**
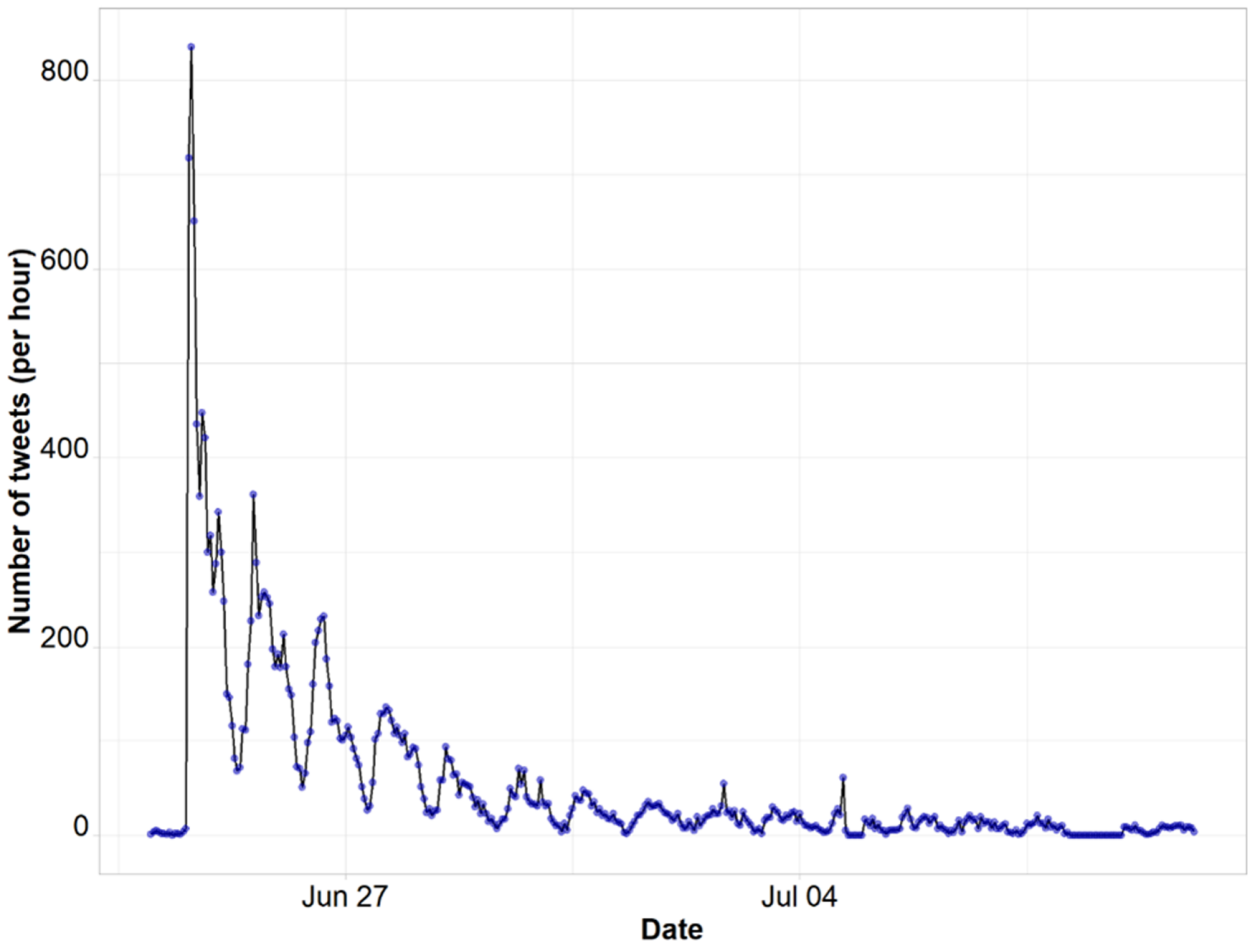
Tweet frequency per hour.

We collected 3,161,353 *Roe vs. Wade* related tweets posted in English language during the entire study period. Of these, a subsample of 23,044 which had mentions related to racial discourse were filtered using the relevant keywords and phrases. After data preprocessing and removal of duplicates, 20,858 unique tweets posted by 17,544 user accounts were retained for analysis. Of the retained accounts, about 3.4% were verified (blue badge next to account user's profile name indicating that an account of public interest is authentic) and about 90.6% sent only one tweet. The average tweet per account was 1.19 (SD = 1.85). The number of followers per account ranged from 0 (one account) to 58,651,318. The median number of followers was 304. The retrieved tweets were retweeted a mean of 3.1 (SD = 68.7) times and given a mean of 13.6 (SD = 319.5) favorites by Twitter users. Figure 1 shows the volume of tweets per hour.

**Figure 2:**
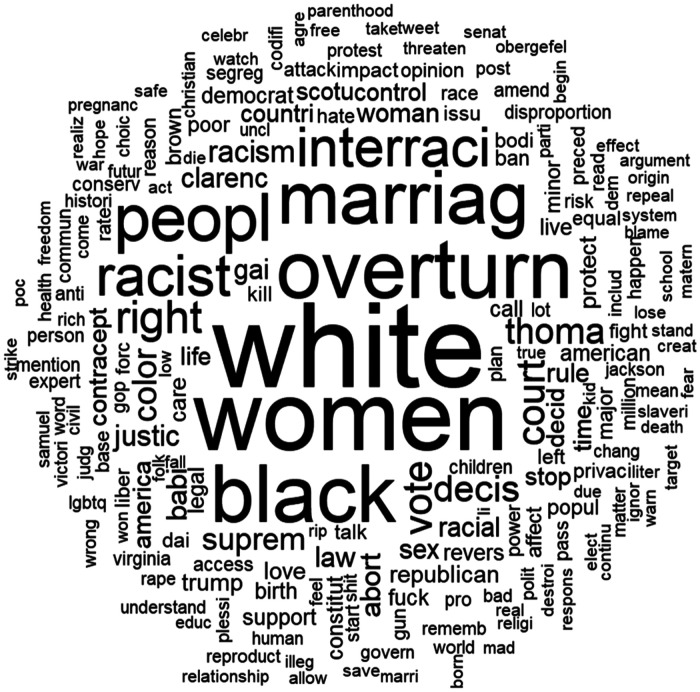
Word cloud of top 200 words (stemmed) related to Roe vs. Wade and race, ethnicity, and racism. Words with larger fonts represent higher frequency in the corpus after preprocessing text.

There was a total of 282,062 unique words and 1,853,293 characters (excluding stop words). The most frequently used words after preprocessing are shown in Figure 2. The most commonly used word was “white” (10,351). The next 19 most commonly occurring stemmed words were “*women*” (8400), “*overturn*” (6662), “*black*” (6470), “*marriag*” (5963), “*peopl*” (5096), “*racist*” (4162), “*interraci*” (4035), “*abort*” (3995), “*right*” (3021), “*vote*” (2462), “*court*” (2400), “*decis*” (2236), “*thoma*” (2139), “*color*” (1913), “*suprem*” (1668), “*racism*” (1579), “*justice*” (1455), “law” (1417) and “*babi*” (1400).

### Sentiment polarity analysis

**Figure 3:**
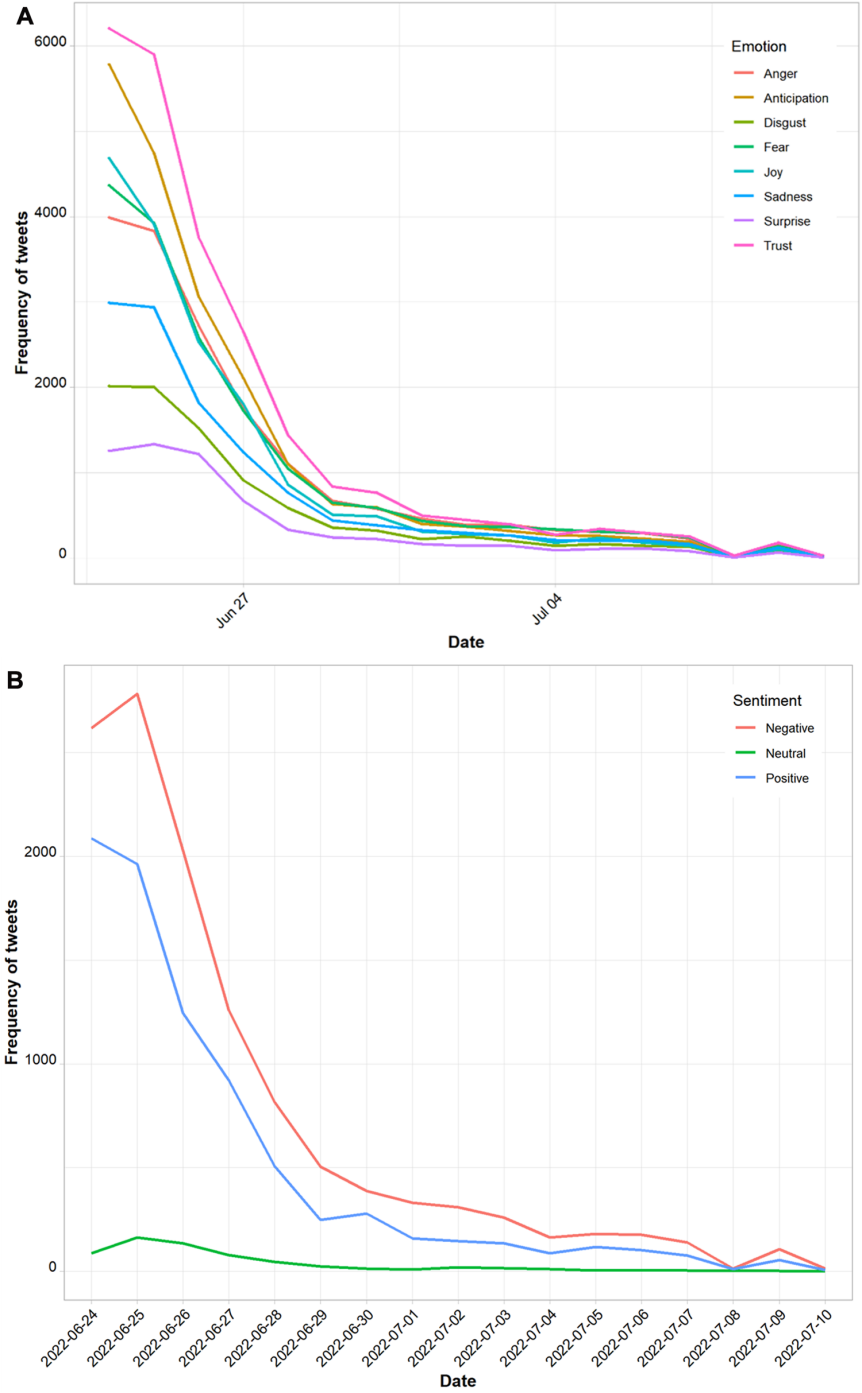
Analysis of (**A**) tweet emotions (anger, anticipation, disgust, fear, joy, sadness, surprise and trust) and (**B**) sentiment polarity over time.

There was an overall negative sentiment polarity in related to racism, race and ethnicity in *Roe vs. Wade* Twitter discourse, with mean polarity scores of −0.4 [standard deviation (SD), 1.5]. Majority of tweets (60.0%, *n *= 12,092) were classified as expressing negative sentiments, with 39.0% (*n *= 8,145) expressing positive sentiments and 3.0% (*n *= 619) expressing neutral sentiments. In addition, tweets with a negative sentiment polarity declined at a slower rate over time (Figure 3B).

### Emotion analysis

Of the eight basic emotions, trust was the most common emotion expressed in approximately one-fifth (*n* = 29,144) of all tweets with themes ranging from the decision impacting more women, children and people of color, being rooted in racism and having repercussions beyond abortion. Examples:

“*This past Friday, Roe fell. This is not the beginning nor the end of the attack on our rights, and we will continue fighting. We trust people who are able to get pregnant to know what is best for their bodies and lives. #whatifdmv #racialjustice #roevwade*”

and

“*@username I agree every woman should have every right to make her own reproductive choices. But it's not about race. Really restrictions to obtaining a safe abortion are going to affect women of colour more, so there's not going to be more white people because Roe was overturned.*”

and

“*This concerns not only the women of US, but all women in the world. Unsafe abortions might increase across the globe, risking many lives. Reversing Roe vs. Wade is also a masked form of racism. Above all,this strengthens the undying culture of sexism. Read* [Link]”

Anticipation was the second most common emotion present in 18.2% (*n *= 25,459) of tweets. Example:

“*It's been in data for decades tho. They knew this was coming. The overturning of roe* vs. *wade was simply symbolic..the overturning of roe* vs. *wade was a pathetic attempt to reverse the pendulum and make whites the majority. Unless you all have cave men on Ice, it’ll never happen*”

and

“*@username @username @username They’re just getting started. Their reaction to COVID Trump, Roe, and the insurrection should alert you that nothing is off the table. Many women will die, blacks and other minorities will be second or third class citizens under their control. It's already begun.”*

The third most common emotion was joy (15.5%) and include topics ranging from saving black lives, highlighting the impact on racial disparities and satisfaction with the Supreme Court's decision. Example:

“*@username I was really pleased to see @username issue an official statement making some of the same structural connections, emphasising also the racially disparate impact. [Link]*”.

and

“*Overturning Roe* vs. *Wade should be a huge victory for black people because 1,200 to 1,500 black babies are aborted everyday and 70 percent of the abortion meals are located in the inner cities and not the suburbs*”

The least common emotions found in tweets were anger (11.6%.), disgust (6.2%) and surprise (4.2%). It is noteworthy that these emotions are indicative of words rather than tweets.

### Structural topic modeling

**Figure 4:**
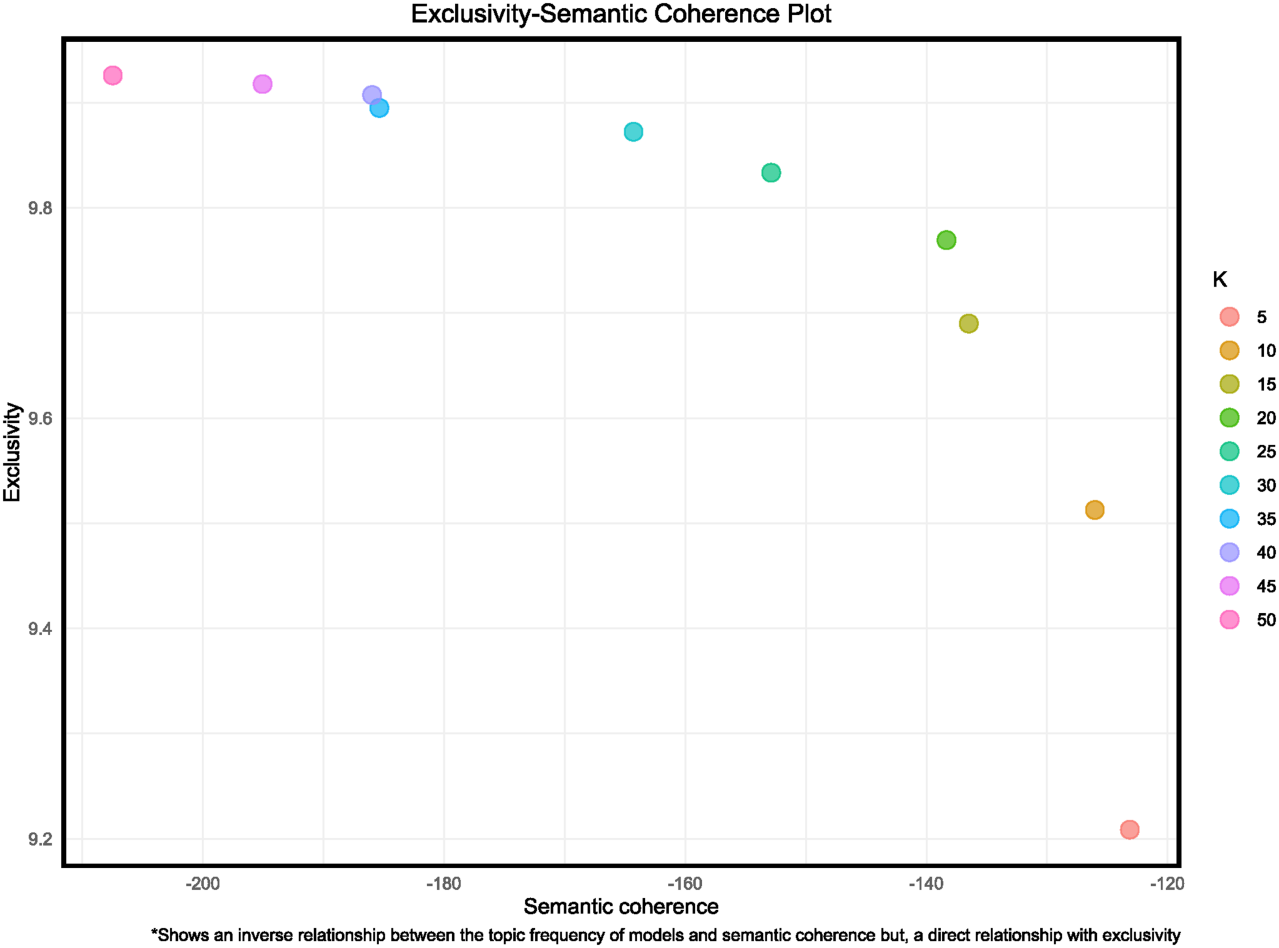
Exclusivity-semantic coherence plot for refined STM model.

This section provides an overview of the output from the trained topic models, results from the STM, themes that are most relevant to the objectives of this study, topic co-occurrence network and topic variations by sentiment polarity. By doing so, we provide nuanced insights into the array of discourses related to race, ethnicity, and racism that users of Twitter generated following the overturn of *Roe vs. Wade*. Figure 4 and Table 1 represent outputs from the training models for the various number of topics. Both show that the model with 20 topics performed well relative to the other models and was chosen as the best-fitting model after validation of the different models.

**Table 1:** Structural topic model diagnostic table.

Number of Topics	Exclusivity	Semantic Coherence	Held-out Likelihood	Residual
5	9.21	−123.13	−5.79	5.58
10	9.51	−126.03	−5.68	4.84
15	9.69	−136.49	−5.65	4.40
20	9.77	−138.34	−5.65	4.10
25	9.83	−152.87	−5.63	3.95
30	9.87	−164.29	−5.63	3.94
35	9.89	−185.37	−5.63	4.23
40	9.91	−185.97	−5.62	3.94
45	9.92	−195.05	−5.62	3.93
50	9.93	−207.47	−5.60	4.33

**Figure 5:**
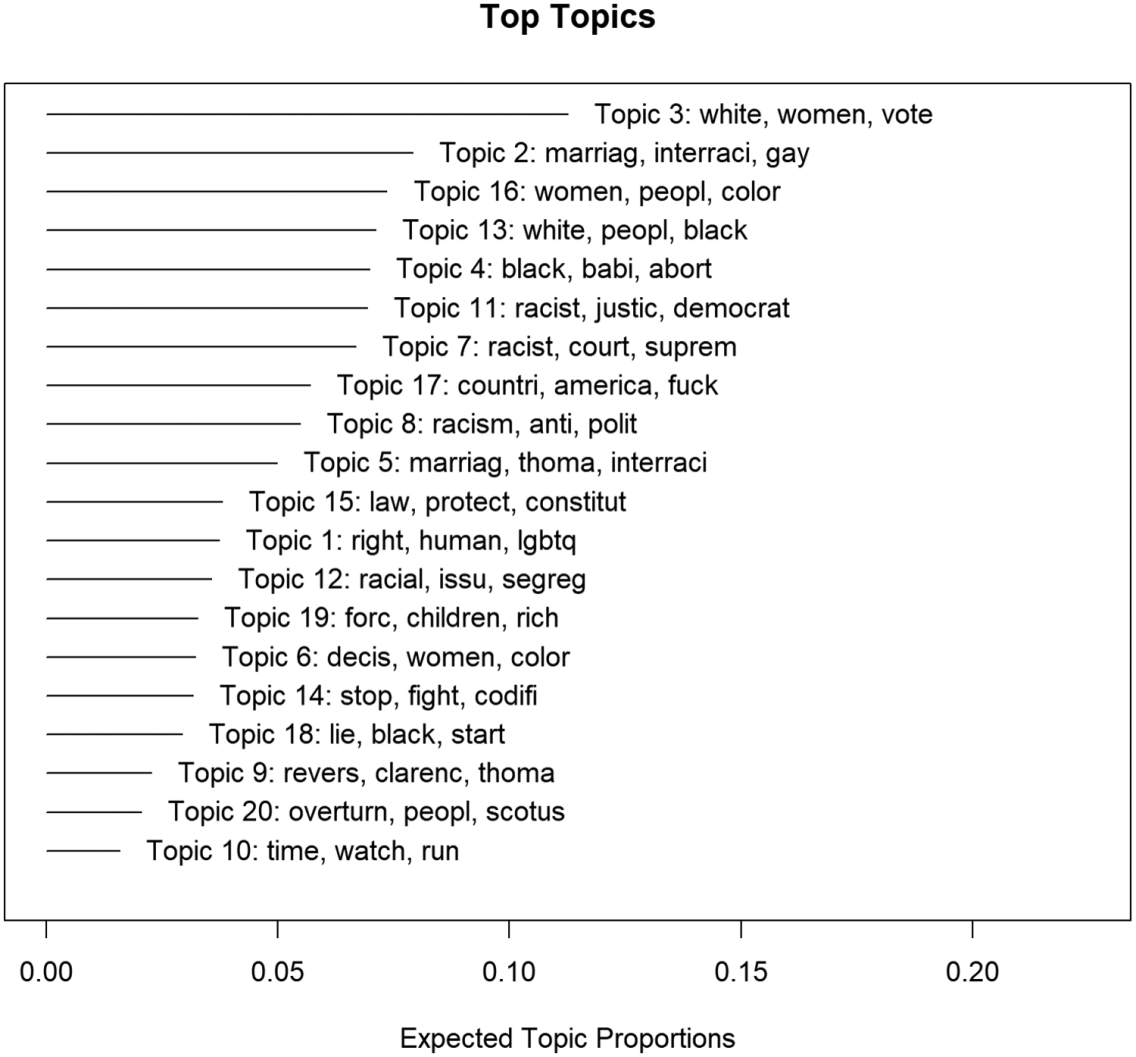
20-topic solution generated by the STM of tweets including the top three associated word stems.

Figure 5 illustrates the 20 latent themes found with the corpus each accompanied by their top three words together with their prevalence in the data. The interpretations of these topics are further listed in the second column of Table 2. The top 10 words for each topic are listed in the fourth column of Table 2. The predominant topics which emerged from the STM were related to “racial resentment” (topic 2, 11.3%), “human rights” (topic 2, 7.9%), and “socioeconomic disadvantage” (topic 16, 7.4%) Together, these topics accounted for approximately one-third of all topics. The topic with the least prevalence was related to “Protestations” (topic 10, 1.6%).

**Table 2:** Themes identified by structural topic modeling.

Topic #	Label	Percentage	Top 10 words	Sample Tweet
3	Racial Resentment	11.27	white, women, vote, overturn, trump, major, minor, republican, power, blame	“@username I mean, also 53% of white women voted for Trump, so… demographically, white women have to own this. Every other category of women showed up and did what they needed to do, but white women voted for this. Without his 3 appointments, we’d still have Roe.”
2	Human Rights	7.91	marriag, interraci, gay, sex, contracept, legal, privaci, overturn, control, ban	“@username @username @username @username @username @username @username It's not but the Roe vs. Wade WAS underpinned by the right to privacy, the scotus rules it's not a protected right in this judgement, that same right to privacy underpins the right to contraception,gay marriage, interracial marriage and was used to repeal sodomy laws.”
16	Socioeconomic disadvantage	7.35	women, peopl, color, affect, overturn, poor, access, communiti, disproportion, die	“imagine someone's tweeting “damn latinx and black ppl are gonna suffer cuz of the Roe vs. Wade overturn” and ur only take away is the use of the word latinx… now imagine if u redirected all that anger towards the word latinx over to real life issues [Link]”
13	Women's Autonomy	7.11	white, woman, overturn, decid, black, bodi, protest, person, choic, peopl	“But passing Roe vs. Wade was quite literally these “old white men” agreeing that no man should decide what a woman does with her body hence why it was passed. Had they rejected it that would have concluded Gilead states get to decide what a woman can do with her body #Backfired [Link]”
4	Black Genocide	6.97	black, babi, abort, popul, plan, million, kill, american, racist, live	“@username @username Lol huh? Bro if anything the black population has slowed it's growth due to Roe vs. Wade. 20million black babies have been killed since 1973 because of Roe vs. Wade. The founder of Planned Parenthood was a known racist and targeted black babies.”
11	Justice Clarence Attacked	6.94	racist, justic, democrat, call, liber, left, thoma, overturn, attack, hate	“@username @username Have you notice they trying to put all these decisions on black men by posting Clarence thomas everywhere. They STILL wana put it on us. And its exactly why white liberals keep losing. #RoeVWade #RoeOverturned”
7	Education	6.68	racist, court, suprem, overturn, life, brown, wrong, plessi, victori, republican	“@username @username @username Both. He's saying that Brown v Board cancelled the precedent set by Pelsey(?) in the same way SCOTUS just got rid of the Roe precedent. But he's doing it for plausible deniability to be racist and speaking to his base while he's doing it. Gaslighting.”
17	Systematic Inequality	5.71	countri, america, fuck, peopl, day, white, care, futur, world, free	“@username @username So sad when the facts are considered hate. You don’t like the fact that for RW extremists the abortion issue started in racism &amp; segregation? It's a fact. They didn’t care when Roe was decided. [Link]”
8	Gun Control	5.03	racism, anti, polit, talk, gun, histori, system, supremaci, overturn, christian	“It’s not too late. History is still unfolding. That said, if you’d told me what lay in wait for my 2017 self (a botched pandemic, an insurrection, Roe gone, anti-anti-racist backlash, metastasizing gun violence, etc.) I would have been skeptical that it could be THAT extreme”
5	Interracial Marriage	4.98	marriag, thoma, interraci, love, justic, overturn, court, clarenc, virginia, rule	“@username And noticeably omits interracial marriage which was not permitted in the Constitution. A later right which he availed of just like Roe and these cases this hypocrite mentioned. [“
15	Equal Protection	3.67	law, protect, constitut, reason, rule, equal, pass, govern, bad, creat	“@username @username @username That’s not what that means. States have had different laws and regulations from other states since our founding. Now if the court had come out and said Roe is the law of the land for black women but it's overturned for whites women that would be an equal protections issue.”
1	LGBTQ Civil Rights	3.53	right, human, lgbtq, take, civil, act, begin, come, step, remov	“If you *like* these times; Roe vs. Wade overturned, trans people hurt and killed, gun rights over school shootings and cop budgets expanded while they keep killing black people, please consider why. Why pain and death of people trying to live their own lives feels like your victory”
12	School Desegregation	3.57	racial, issu, segreg, educ, school, gender, inter, public, religi, social	“Some of the impacts from the end of #Roe:—Limits of education &amp; career advancements—Ongoing systemic racism—Criminalization of pregnancy—Maintaining gender inequality—Ongoing poverty cycle”
19	Forced Pregnancy	3.41	forc, children, rich, white, support, kid, birth, famili, pay, child	“Politicians care more about money than your rights. Both sides want to keep the debate alive because it rakes in $$$. The fight around Roe is more insidious than religion or life. It's another example of political greed at the expense of women, especially women of color.”
6	Disproportionate Harm	3.21	decis, women, color, impact, expert, post, threaten, destroy, warn, reproduct	“How outlawing abortion will worsen America's maternal mortality crisis. The SCOTUS decision will disproportionately impact Black women, who are 3× more likely to die during pregnancy or childbirth than whites. Blacks are also more likely to be uninsured. [Link]”
14	Codifying Roe	3.16	stop, fight, codifi, dem, slaveri, won, elect, war, kill, biden	“SCOTUS enshrining minority rule for the white evangelical snowflakes. Let's call all white people minorities now. They are acting like such fragile babies they have to hide behind such the SCOTUS religious minority protection. [Link]”
18	Rape Claim	2.93	lie, black, start, pro, rape, base, claim, told, friend, girl	“The overturning of Roe vs. Wade was influenced by fears that white people are becoming the minority. They’re terrified of losing political power and banning abortion to increase the “domestic supply” of white infants is their solution. [Link]”
9	Jackson Rips Clarence	2.19	evers, clarenc, thoma, risk, uncl, interraci, marriag, jackson, samuel, rip	“Please know, the Democrats had plenty of time to codify Abortion with a constitutional amendment, but knew it would never pass. They relied on a poorly scripted SCOTUS judgment (of white men). She's trying to satisfy the base while admitting #RoeVWade had no legal standing. [Link]”
20	Liberalism & Abortion	2.05	overturn, peopl, scotus, white, make, support, real, happen, busi, week	“I ironically find myself without a lot to say on #Roevs.Wade / #DobbsVsJackson. I think this ruling is a loss for #womensrights. At the same time, what does centrist liberalism expect when it can’t even have a discussion about how #MargaretSanger hated black people? ”
10	Protestations	1.59	time, watch, run, mention, video, suprem, minut, forgot, apolog, beg	“I can’t help but see how folks are responding to the overturning of Roe vs. Wade like “this is too far.”…..so is y’all saying antiblack racism is acceptable? The interest convergence with white liberal prerogative isn’t lost on me…..#Roevs.Wade”

**Figure 6:**
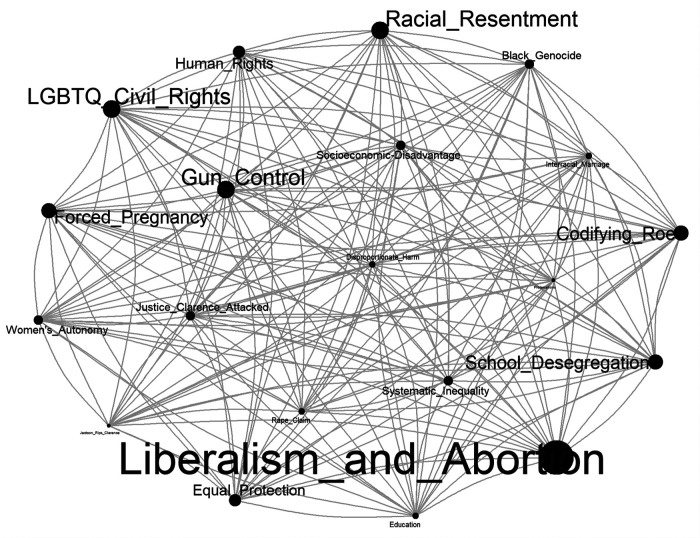
Network analysis for all co-occurring topics from the STM.

Figure 6 is a co-occurrence network analysis which depicts the frequency with which a specific topic occurred with other topics on the corpus of tweets and thus enabling examination of several pairs of co-occurring topics simultaneously. Each topic observed with one or more co-occurrence was plotted and represented by word tags referred to as nodes. Larger nodes depict topics with a higher frequency co-occurrence in the corpus while smaller nodes represent topics which co-occurred less frequently. Furthermore, nodes in close proximity with each other are connected directly or indirectly implying that they were mentioned together, have similar neighbors, or are connected together by other nodes. On the other hand, nodes distant from each other are less connected which suggest that they are mentioned less frequently and may not be directly or indirectly connected by a neighbor. Also, topics are connected by connecting lines (edges) with the width of each line directly proportional to the number of times a connection of observed. The complete network of connections comprised 20 interconnected nodes (i.e., topics) and 400 edges (i.e., interconnecting lines between nodes).

**Figure 7:**
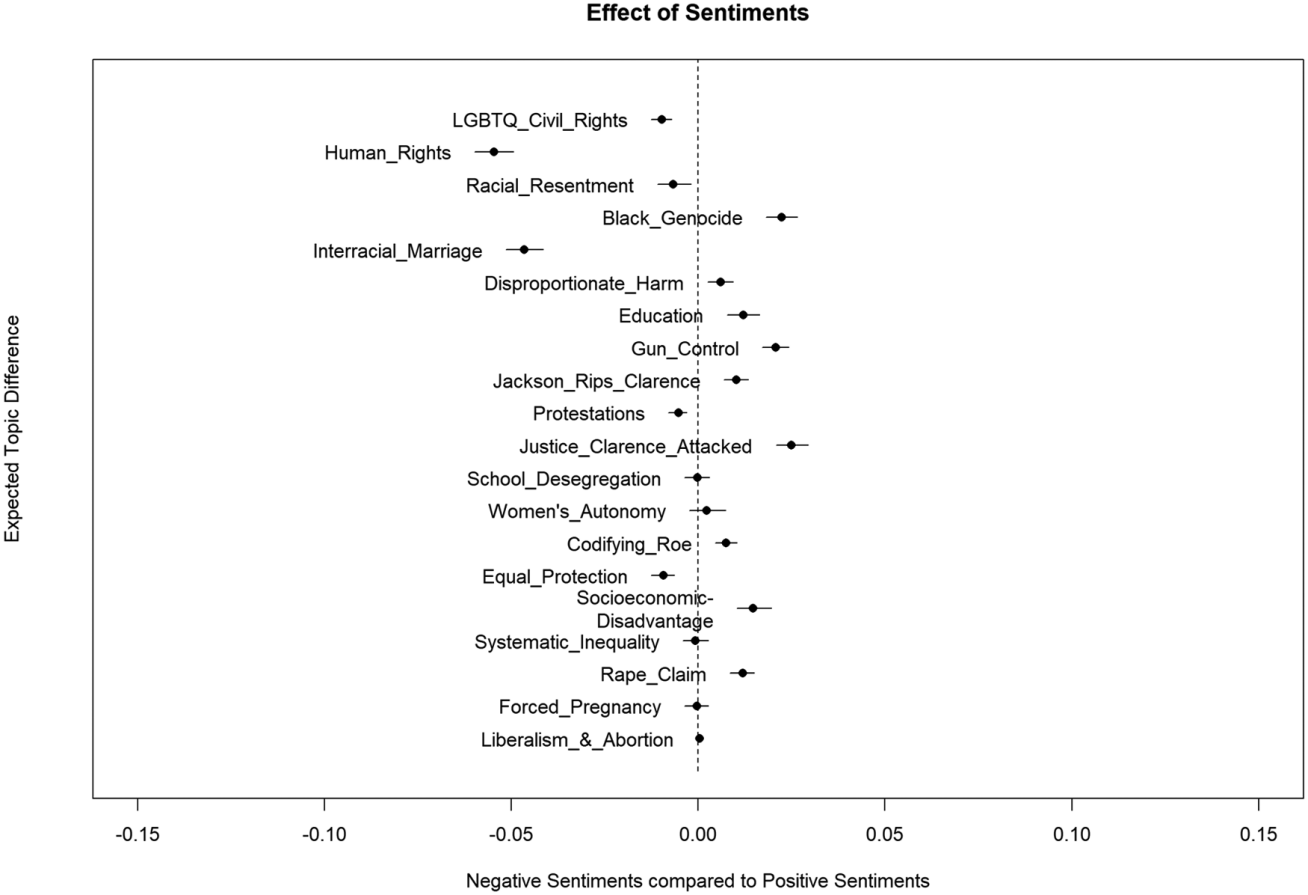
Difference in topic prevalence by sentiment polarity.

Figure 7 is a graphical illustration of the relationships between the between the 20 topics and sentiment polarity based on the expected topic proportions with the panel divided by the zero value of the beta coefficient on the x-axis representing a neutral line. The dots represent point estimates (mean values) for the difference between topic prevalence for the positive and negative sentiments while the lines bracketing the dots indicate the 95% confidence intervals (CIs) of the differences. Confidence intervals that include 0.0 are considered not statistically significant.

Topics on the left of the zero line indicated topics associated with negative sentiments while topics to the right of the zero line indicate topics that were associated with positive sentiments. The STM found a significant association between positive sentiments and topics related to Black genocide, disproportionate harm, gun control, Jackson attacking Clarence, vitriolic and racial attacks on Clarence, codifying Roe, socioeconomic disadvantage and rape claim. In contrast, LGBT rights, human rights, racial resentment, interracial marriage, protestations and equal protection all exhibited a significant association with negative sentiments.

## Discussion

This study extends abortion public opinion literature in several ways. By employing computational methods, this paper used multiple methods of inquiry (topic modeling, sentiment, and emotion analyses) to understand public opinions about race, ethnicity, and racism in Twitter conversations related to the reversal of *Roe vs. Wade*. Firstly, this study demonstrates the utility of user-generated content from social media as an important tool for the rapid assessment of public reactions to changes in reproductive health legislation. Secondly, it provides a nuanced understanding of the varied perspectives regarding the links between race/ethnicity and racism and the reversal of *Roe*. Thirdly, it extends the extant but limited literature on abortion restrictions and racial and ethnic disparities.

The sentiment analysis revealed that majority of the tweets identified and analyzed in this study expressed predominantly negative sentiments. On the other hand, emotion analysis demonstrated a dominant pattern of tweets linked mainly to trust (∼20%), anticipation and joy. There were 20 topics identified from our structural topic modeling were related to ethno-racial discourses in post-*Roe* related tweets. Of these, ten that were classified as having expressed positive sentiments, seven contained tweets expressing negative sentiments, and three themes neither explicitly expressed positive nor negative sentiments. Overall, the topics uncovered in the discourses were predominantly related to racial resentment, human rights, socioeconomic disadvantage, women's autonomy, and black genocide. Several studies have also used social media data to examine perceptions related to the reversal of *Roe vs. Wade*. One study examining temporal trends in public perceptions on Twitter to the overturning of *Roe vs. Wade* showed that towards the year 2022, sentiments became increasingly negative and less neutral and positive (2). However, despite the large sample of tweets collected over time, this study did not examine racial discourses. Another study (4) which used Twitter content to examine backlash to Georgia's abortion ban also showed that concerns regarding race, minorities, and immigrants featured during the discourse further demonstrating concerns about the links of abortion restrictions with racial disparity and thus, a consistent subject of public interest with abortion bans. While limited by a small sample size, this study did not also examine sentiments associated with tweets. Overall, the findings from these studies therefore raises the question about whether and, to what extent the findings of negative sentiments are related specifically to racial concerns or generally to the Supreme Court's decision.

Our natural language processing (*nlp*) approach underscores that there is some evidence that Twitter users linked the Supreme Court ruling in *Dobbs*, and probably other state-level abortion restrictions, to race, ethnicity, and racism. According to the literature on abortion restriction, the plausibility of this link relates to evidence that suggests that Black women are more likely to seek an abortion and consequently, are more likely to be more adversely impacted by policies restricting access to safe and legal abortion (47). Given the relative importance of access to abortion care, the predominance of negative sentiments expressed in tweets in this study, though not surprising, can be explained by the psychological reactance theory. According to this theory, individuals would express motivation to restore specific behavioral freedoms (real or perceived) whenever these freedoms are threatened or eliminated (48). This motivation (reactance) comprises negative feelings such as the expression of arguments against freedom restrictions as well as negative cognitions. Drążkowski and Trepanowski (49) while employing this theory in their study showed an increased reactance following Poland's abortion rights restriction. The finding of “joy” in the sentiment analysis of discussions of abortion access and race may highlight the positive emotions expressed by some individuals in relation to the reversal of *Roe vs. Wade* possibly because the decision aligns with their moral beliefs and values. It is however important to note that the presence of “joy” in a sentiment analysis does not necessarily imply that the sentiment is universally positive or that everyone is experiencing joy as there are likely to be a range of emotions and perspectives regarding this the issue, including those who are deeply concerned or upset by the reversal of *Roe vs. Wade*. A thorough analysis from real-world observational studies that takes into account multiple factors such as moral beliefs and values of different groups, as well as the potential impact of the decision on women's health and well-being would further strengthen the findings from this study.The prevalent negative sentiments together with reactance as shown in our study are reflected in the topics related to racial resentment and protestations which emerged from the topic modeling analysis. Also, findings from the STM showed that within the ethno-racial discourse on Twitter related to the fall of *Roe*, themes related to the LGBTQ rights, interracial marriage, equal protection, systematic inequality and gun control emerged. The emergence of these themes could be an indication of the perceived aftermath that is likely to follow *Dobbs*.

As the Supreme Court's ruling in *Dobbs* creates uncertainties for racial inequalities amongst other concerns, our findings reiterate the need for more evidence to determine how policies restricting legal abortion might affect access to abortion services by women of color. Furthermore, while it is expected that future studies will examine real-world repercussions of *Roe vs. Wade* on racial disparities, this study helps to lay the foundation upon which these studies can leverage to evaluate whether and, to what extent early public concerns as expressed on social media reflect individual experiences and in addition, will provide further validation regarding the utility of social media data for investigating public perceptions of social and health-related phenomena including reproductive health.

There are several limitations of this study. Data for this study was obtained from Twitter. While it is one of the most popular microblogging platforms, the findings from this study may not be representative of reactions or perceptions on other social media platforms. In addition, tweets used for analysis may not be representative of the entire US population given the demographic and geographic diversity of Twitter users. However, the extent of the diversity is sufficient to capture public concerns that could be used to inform future research. Also, because Twitter demographic data does not include race, it was not possible to examine how sentiments and topics varied by race/ethnicity which could have been valuable to the findings in this study. Second, this study only used tweets posted in English language thus leaving out a proportion of tweets expressed in other languages (for example, Spanish), especially with the growing population of Latinx population in the US (50) which would have generated findings relevant to the conclusions in this study. A third limitation is related to the keywords used to retrieve *Roe vs. Wade* related tweets. Although wildcards for one or any of the queries can help capture misspellings, we did not, however use this in our study. This would have increased the sample size for this study and in turn, provide additional information relevant to the objectives of this study. Finally, this study focused on Twitter reactions after the overturn of *Roe* and hence was not able to examine the evolution of sentiments and themes before the Supreme Court draft opinion was leaked and the reactions after it was leaked before the official reversal of *Roe*.

## Conclusion

User-generated content from Twitter can be leveraged to monitor reactions to changes in reproductive health policies and legislations. Th*e* use of natural language processing to perform text analysis of tweets posted in response to the reversal of *Roe* revealed the dynamic nature of sentiments and several themes which emerged in Twitter discourse following the federal abortion restriction. The findings from our study illustrate a wide range of ethno-racial concerns following the reversal of *Roe*. With available evidence suggesting that only socially-advantaged women had limited access to abortion services in the pre-*Roe* era, our study strengthens the need for ongoing surveillance of racial and ethnic disparities in abortion access in the post-*Roe* era.

## Data Availability

The original contributions presented in the study are included in the article/supplementary materials, further inquiries can be directed to the corresponding author/s.
